# Cancer incidence and mortality in the municipality of Pasto, 1998 - 2007

**Published:** 2012-12-30

**Authors:** María Clara Yépez, Luis Eduardo Bravo, Arsenio HidalgoTroya, Daniel Marcelo Jurado, Luisa Mercedes Bravo

**Affiliations:** a Centro de Estudios en Salud, Universidad de Nariño, Cancer Registry of Pasto. E-mail: macych@gmail.com; b Departament of Pathology, Universidad del Valle, Colombia. E-mail: bravo.luiseduardo@gmail.com; c Cancer Registry of Pasto, Departament of Mathematics, Universidad de Nariño. E-mail: arsenio.hidalgo@gmail.com

**Keywords:** Incidence, mortality, cancer, prevention, *Helicobacter pylori*, human papillomavirus

## Abstract

**Introduction:**

In Colombia, information on cancer morbidity at the population level is limited. Incidence estimates for most regions are based on mortality data. To improve the validity of these estimates, it is necessary that other population-based cancer registries, as well as Cali, provide cancer risk information.

**Objective:**

To describe the incidence and cancer mortality in the municipality of Pasto within the 1998-2007 period.

**Methods:**

The study population belongs to rural and urban areas of the municipality of Pasto. Collection, processing, and systematization of the data were performed according to internationally standardized parameters for population-based cancer registries. The cancer incidence and mortality rates were calculated by gender, age, and tumor

**Results:**

During the 1998-2007 period 4,986 new cases of cancer were recorded of which 57.7% were in female. 2,503 deaths were presented, 52% in female. Neoplasm-associated infections are the leading cause of cancer morbidity in Pasto: stomach cancer in males and cervical cancer in females.

**Discussion:**

Cancer in general is a major health problem for the population of the municipality of Pasto. The overall behavior of the increasing incidence and cancer mortality in relation to other causes of death show the need to implement and strengthen prevention and promotion programs, focusing especially on tumors that produce greater morbidity and mortality in the population.

## Abstract (es)

**Introducción:**

En Colombia es limitada la información de morbilidad por cáncer dentro de la población. Las estimaciones de incidencia para la mayoría de las regiones se basan en los datos de mortalidad. Para mejorar la validez de estas estimaciones, es necesario que otros Registros poblacionales de cáncer, además de Cali, proporcionen información del riesgo de cáncer.

**Objetivo:**

Describir la incidencia y mortalidad por cáncer en el municipio de Pasto durante el período 1998-2007.

**Metodología:**

La población objeto de estudio pertenece al área rural y urbana del municipio de Pasto. La recolección, procesamiento y sistematización de los datos se realizó de acuerdo con parámetros estandarizados internacionalmente para Registros poblacionales de cáncer. Las tasas de incidencia y mortalidad se calcularon por género, edad y localización del tumor.

**Resultados:**

En el período 1998-2007 se registraron 4.986 casos nuevos de cáncer, de ellos, 57,7% se presentó en mujeres. Se reportaron 2.503 muertes, 52% en mujeres. Las neoplasias asociadas con infecciones son la primera causa de morbilidad por cáncer en Pasto: cáncer de estómago en hombres y cáncer de cuello uterino en mujeres.

**Discusión:**

El cáncer, en general, constituye un importante problema de salud para la población del municipio de Pasto. El comportamiento global de la incidencia y el aumento de la mortalidad por cáncer en relación con otras causas de muerte, evidencian la necesidad de fomentar y fortalecer programas de promoción y prevención, enfocados especialmente hacia los tumores que producen mayor morbi-mortalidad en la población.

## Introduction

Cancer is one of the main causes of morbidity and mortality in the world; for 2008 a total of 12,662,600 new cases were estimated of which 52% occurred in males. It is considered that cancer is responsible for 13% of the world's deaths. By 2030, the World Health Organization (WHO) expects significant growth in the magnitude of this important public health problem, as a product of the population's demographic growth and aging. It is estimated that the new cases will rise to 21-million and mortality due to cancer will increase to 45%, causing approximately 13-million deaths. The first causes of morbidity and mortality due to cancer in the world are lung, breast, colorectal, stomach, liver, and prostate cancer.^1^ ^,^ ^2^

In Colombia, information on cancer is still limited at the population level and estimations of incidence are obtained based on mortality data.^3^For the 2002 - 2006 period, 70,000 cases were estimated, with incidence rates per 100,000 person-years, age-adjusted to the world standard population, of 186.6 in males and 196.9 in females. The most frequent site of cancer in males were prostate, stomach, and lung; in females, breast, cervix, and thyroid. The departments with the highest incidence were: Risaralda, Caldas, Antioquia, Valle del Cauca, and Quindío^4^.

To address this problem, the Ministry of Health and Colombia's National Cancer Institute (NCI) launched the Plan for Cancer Control, seeking to set into motion actions by the state, inter-sector actions, business social responsibility, and individual responsibility to provide cancer control and position cancer within the nation's public agenda as a public health problem in Colombia. To execute this plan, it is initially fundamental to describe the epidemiological situation of cancer within the population and evaluate the determinant factors, thus, permitting the healthcare system to guide the oncology services and present an adequate social response.

The Cancer Registry of Pasto (RPCMP, for the term in Spanish) has been continually operating since 1998 and its mission is that of providing quality and reliable information on cancer in the Municipality; thereby, facilitating the implementation of intervention programs aimed at diminishing the burden of this disease in the region. Currently, it is part of the International Association of Cancer Registries (IACR) and the Network of Population-based Cancer Registries of Colombia, along with the Registries in Cali, Bucaramanga, Manizales, and Barranquilla.

This study describes cancer incidence and mortality in the municipality of Pasto during the 1998-2002 and 2003-2007 periods, as the contribution made by the RPCMP to the knowledge of cancer behavior in the Municipality and the contribution to achieving the objective goals of the National Plan National for Cancer Control.

## Materials and Methods

**Figure 1:**
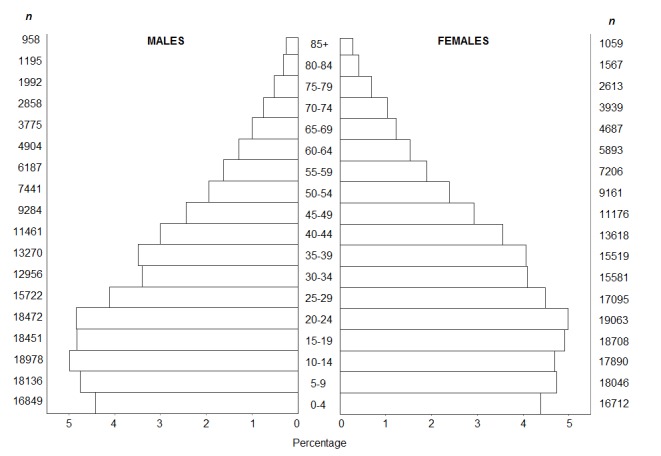
Municipality of Pasto, Colombia. Population structure by sex and 5-year age groups. 2005 General census. Source: Departamento Administrativo Nacional de Estadísticas (DANE). Census population

The study was carried out under a descriptive epidemiological design on cancer in the urban and rural population of the municipality of Pasto, in the Department of Nariño. The population reported for this municipality by Colombia's National Administrative Department on Statistics (Departamento Administrativo Nacional de Estadísticas - DANE) through the last official census of 2005, was 382,422 inhabitants; 81.7% living in the urban zone (Fig. 1).

The study included all malignant tumors diagnosed in individuals residing in the municipality of Pasto during the 1998-2007 period. Additionally, tumors of uncertain nature, benign tumors of the central nervous system (SNC), and tumors *in situ* were also included. Date of incidence corresponds to the first chronological event of diagnostic confirmation of the disease or, in case of the lack of data, date of death was used.

The information was gathered in active, continuous, and systematic manner in the healthcare institutions that generate information on cancer: hospitals, clinics, oncology units, pathology and hematology laboratories, medical centers, specialized clinics, the Municipal Secretary of Health, which is the organism in charge of processing the municipality's death certificates, and DANE, which provides the official mortality base. The data gathered are related to the patient's socio-demographic variables, clinical aspects of the tumor, and follow up. Information collected was worked on according to confidentiality criteria imposed by the International Agency for Cancer on Research (IACR) for Population-based Cancer Registries.

The cases were entered into the system to eliminate duplicates, processing and complementing data. Identification of multiple primary tumors followed IARC standards^5^. The topography (sites) and morphology (histology) of the tumors were coded with the International Classification of Diseases for Oncology Third edition (ICD-O-3). For comparative purposes, the conversion of the sites coding was made to ICD-10 (International Classification of Diseases 10th edition) and some sites were grouped. To validate the internal consistency among variables, an automatic check was made with the IARCcrg Tools program version 2.05 and rare cases were resolved in a scientific committee conformed by specialists.

For the 1998-2007 period, the percentage of cases diagnosed through microscopic verification (histology of primary tumor, cytology, and bone marrow aspirate) in males was 78.3% and 91.9% in female; the percentage of cases registered as death certificate only was 11.5% in males and 10.2% in females. Other diagnostic methods included: imaging, exploratory surgery, clinical impressions, and endoscopy. The percentage distribution of indices of data quality for the first ten sites is shown in Table 1. The percentage of deaths with age unknown was 0.3% and the percentage of deaths with unknown primary site (C76-C80) was 5%.

**Table 1.**
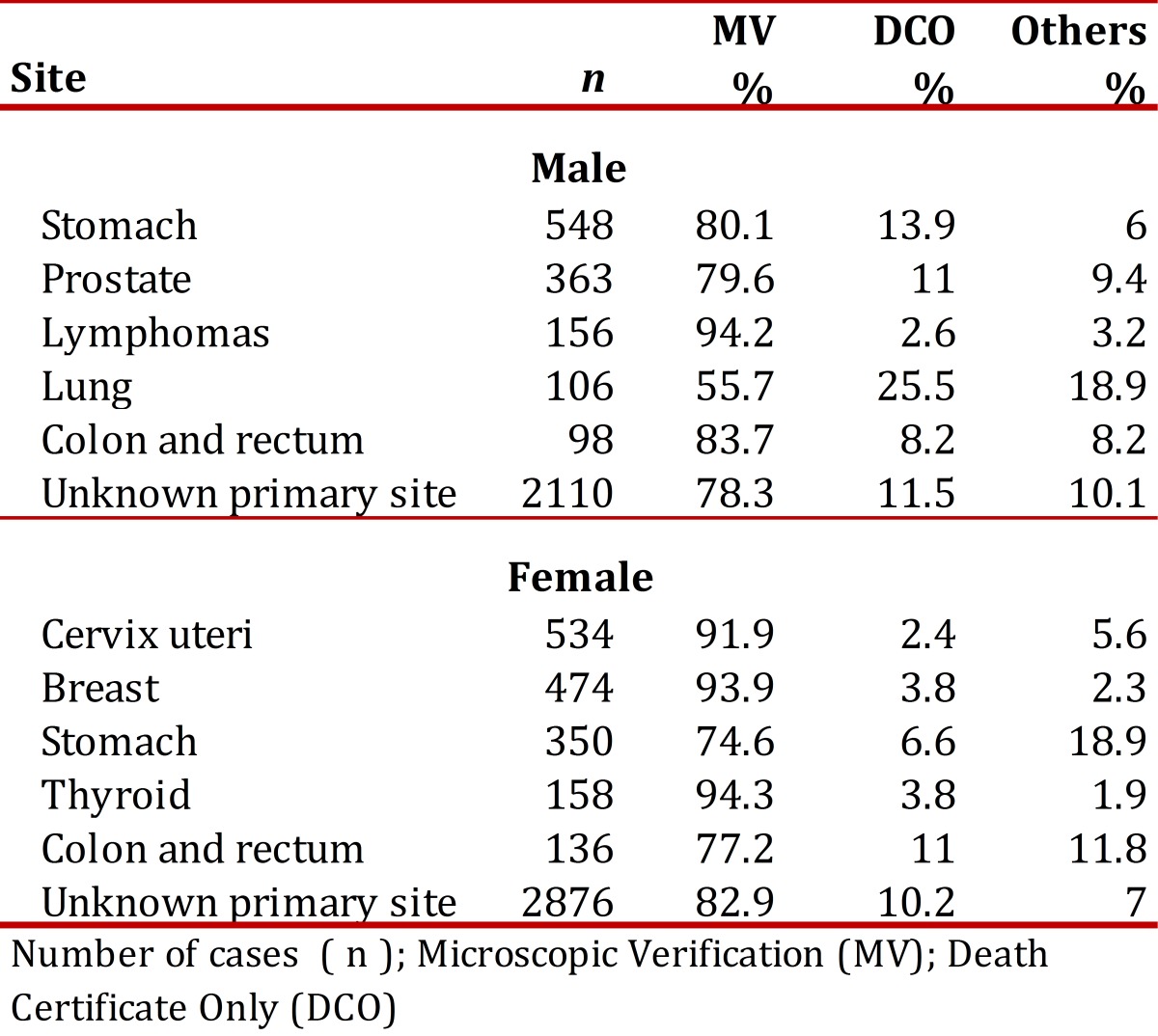
Municipality of Pasto, Colombia. Indices of data quality for the top 5 cancer sites. males and females, 1998-2007, number cases (n);microscopic verification (%MV); Death Certificate Only (%DCO).

Incidence rates and mortality were conventionally calculated by using as denominator the population estimations and projections by midyear calculated from the 2005 official census^6^. Cases without age, basal cell and squamous cell carcinomas of the skin, and tumors *in situ* were excluded. Age-standardized rates were estimated via the direct method with the world standard population, specific rates were calculated via the following variables: gender, sites, and age according to quinquennial ranges (18 categories) and large groupings. Cancer incidence and mortality results are presented for the 1998-2002 and 2003-2007 periods in specific tables or figures and in-depth analysis was performed of the main sites, given that these are diseases of great relevance for the region. To observe the cancer incidence and mortality increase or decrease between both study periods, the rate percentage change was assessed.

## Results

### Global cancer incidence and mortality per 100,000 person-years

During the 1998-2007 period, 4,986 new cancer cases were registered in the municipality of Pasto, of them 57.7% occurred in females. A total of 45.5% of the cases occurred in individuals older than 65 years of age; 2% of the cases were observed in individuals younger than 15 years of age. The mean age upon diagnosis was 62 years of age in males and 57 years of age in females. During the same period, 2,503 deaths occurred due to cancer, 52% occurred in females. The greatest proportion of deaths happened as of 65 years of age, both in males (62%) as in females (53%). The mean age of death for males was estimated at 66 years and 64 years for females.

The number of new cases for the 2003-2007 period (2,640) increased with respect to the number of cases for the 1998-2002 period (2,346). The global rate percentage change of cancer incidence in males was at 5% and 1% in females. The risk of developing cancer was higher in females with a male/female gender ratio (GRm/w) of 0.9/1 during both periods. Tables 2 and 3 describe the number of cancer cases and incidence rates according to gender and sites of the tumor for both periods evaluated.

In the municipality of Pasto, during 1998-2002, cancer was the third cause of death after mortality due to cardiovascular disease and deaths due to external causes. During this period, of a total of 7,784 deaths due to all causes, 1,154 died due to cancer (15%). From 2003 to 2007, this value increased to 1,349 deaths that represented 18% of a total of 7,565 deaths; during this period, cancer became the second cause of mortality in the municipality. The rate percentage change of mortality due to cancer between both periods was 3% in males and 9% in females. Unlike morbidity, the risk of dying due to cancer was similar between males and females (GRm/w of 1:1). Tables 4 and 5 show the number of deaths and rates of mortality by gender and sites for the study periods. The ratio between incidence rates and mortality was 2:1, for both five-year periods for males and females.

**Table 2:**
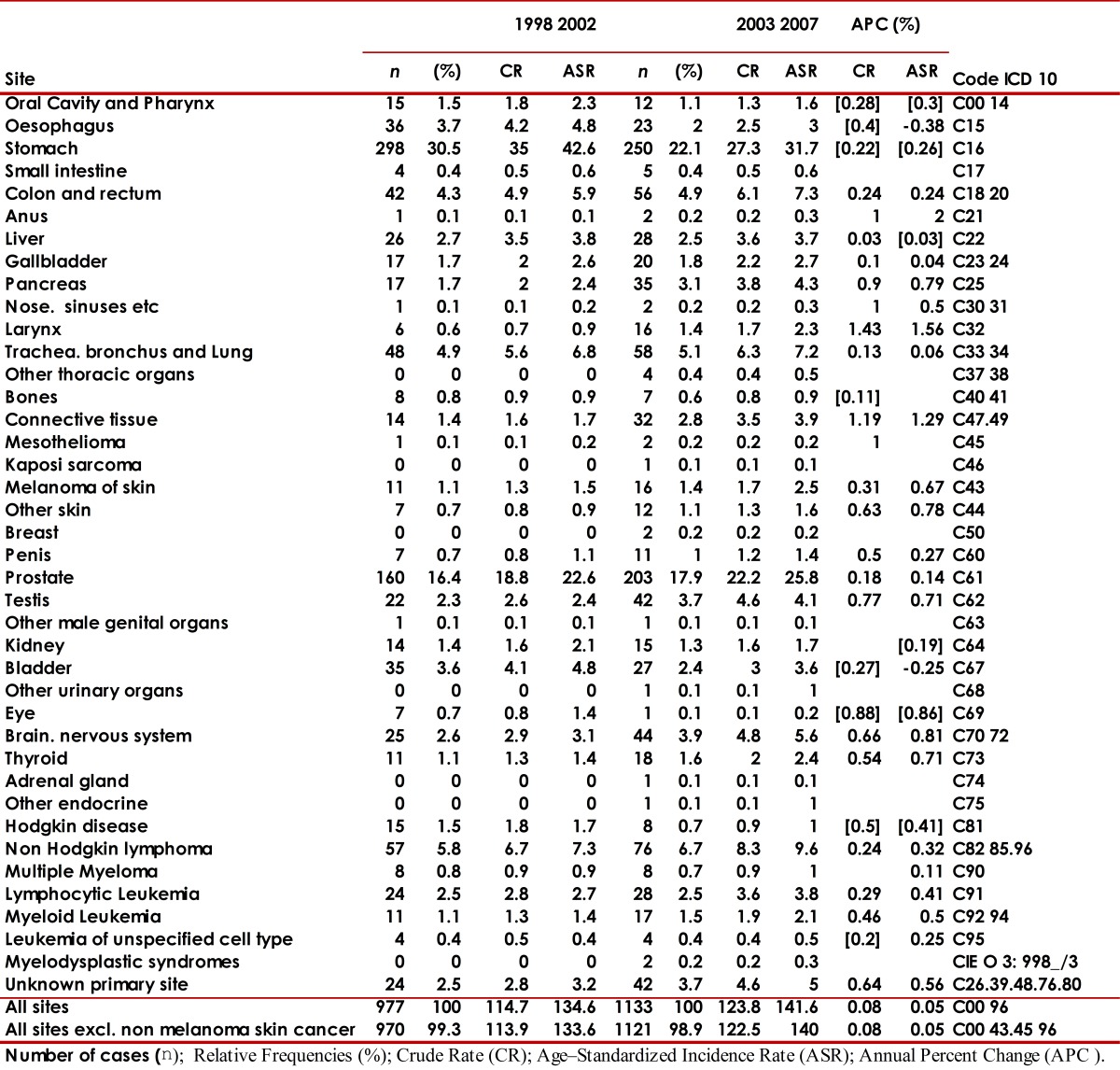
Municipality of Pasto, Colombia. Cancer incidence rates, crude and age-standardized (World standard population) per 100,000 person-years by primary cancer site in males, 1998-2002 and 2003-2007 Number cases (n); Relative Frequencies (%); Rrude Rate (CR); Age - Standardized Incidence Rate (ASR); Annual Percent Change (APC %).

**Table 3.**
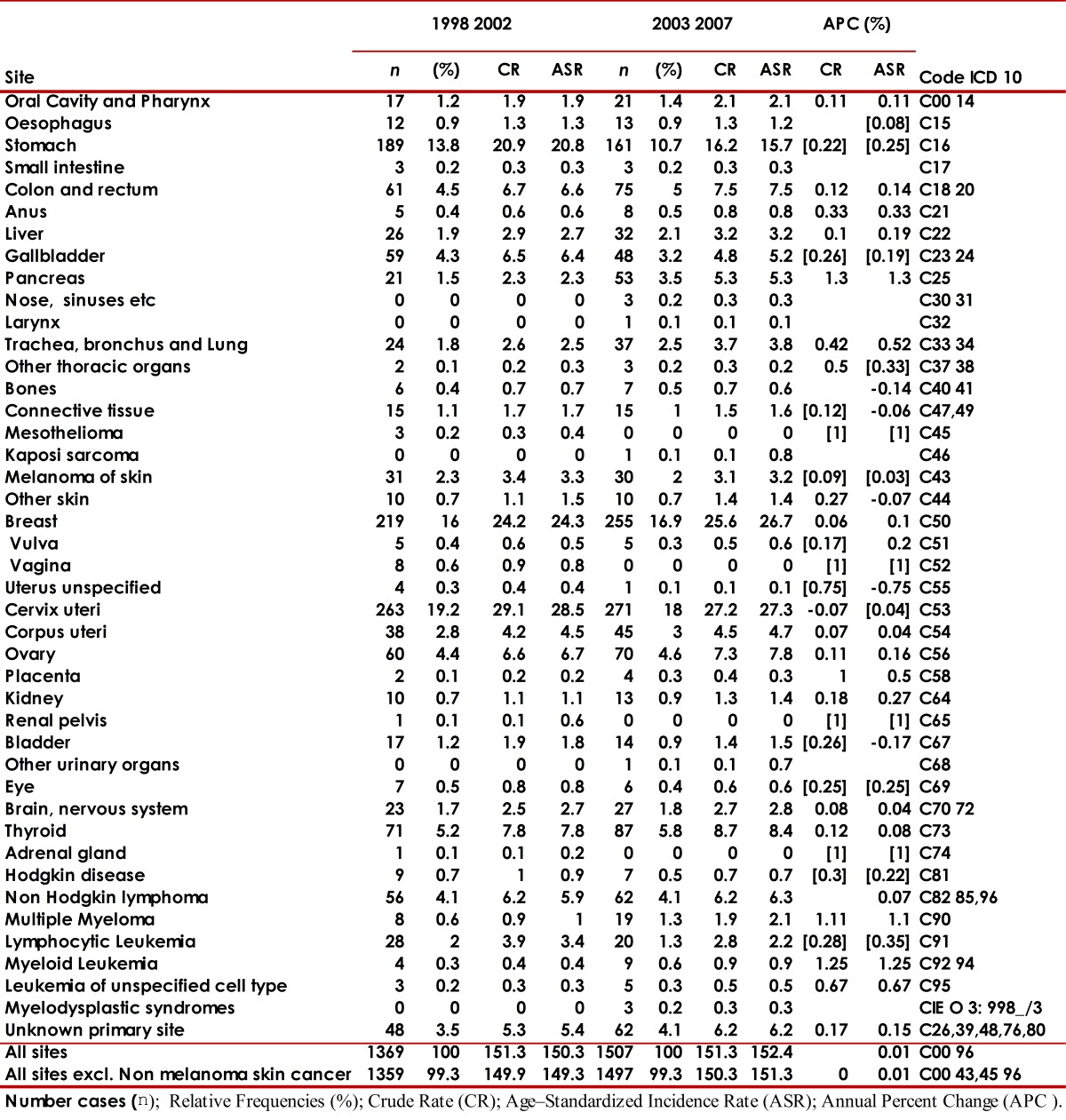
Municipality of Pasto, Colombia. Cancer incidence rates, crude and age-standardized (World standard population) per 100,000 person-years by primary cancer site in females, 1998-2002 and 2003-2007. Number cases (n); Relative Frequencies (%); Rrude Rate (CR); Age - Standardized Incidence Rate (ASR); Annual Percent Change (APC %).

**Table 4:**
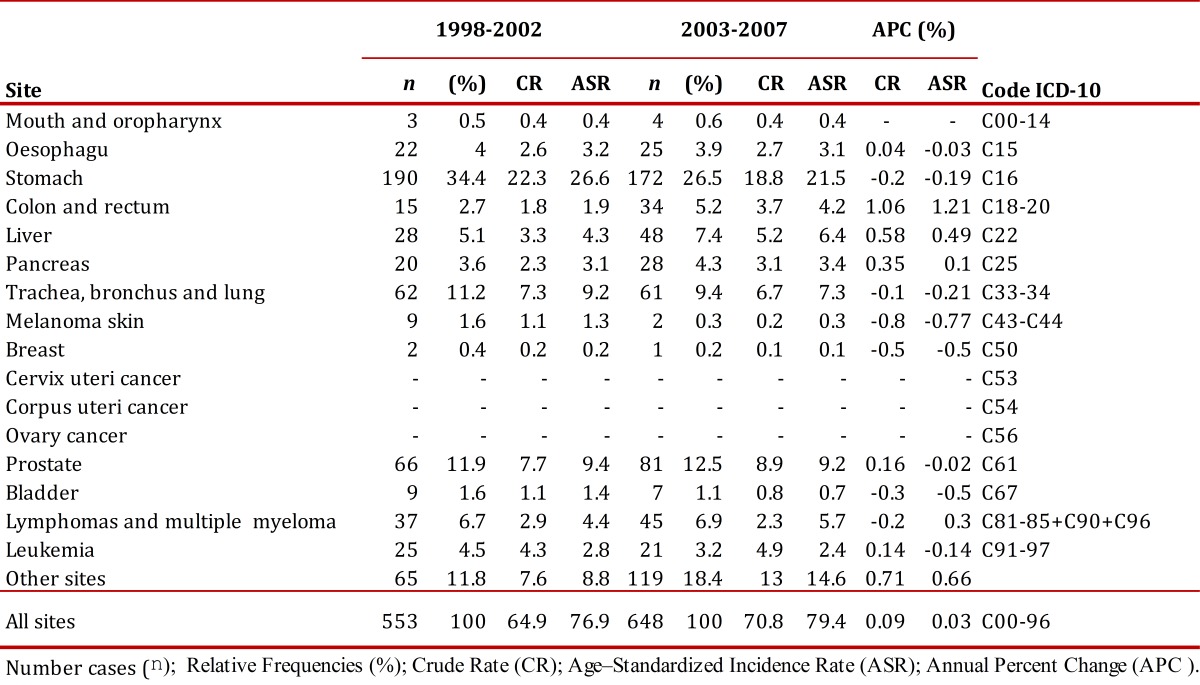
Municipality of Pasto, Colombia. Cancer mortality rates, crude and age-standardized (World standard population) per 100,000 person-years by primary cancer site in males, 1998-2002 and 2003-2007. Number cases (n); Relative Frequencies (%); Rrude Rate (CR); Age - Standardized Incidence Rate (ASR); Annual Percent Change (APC %).

**Table 5:**
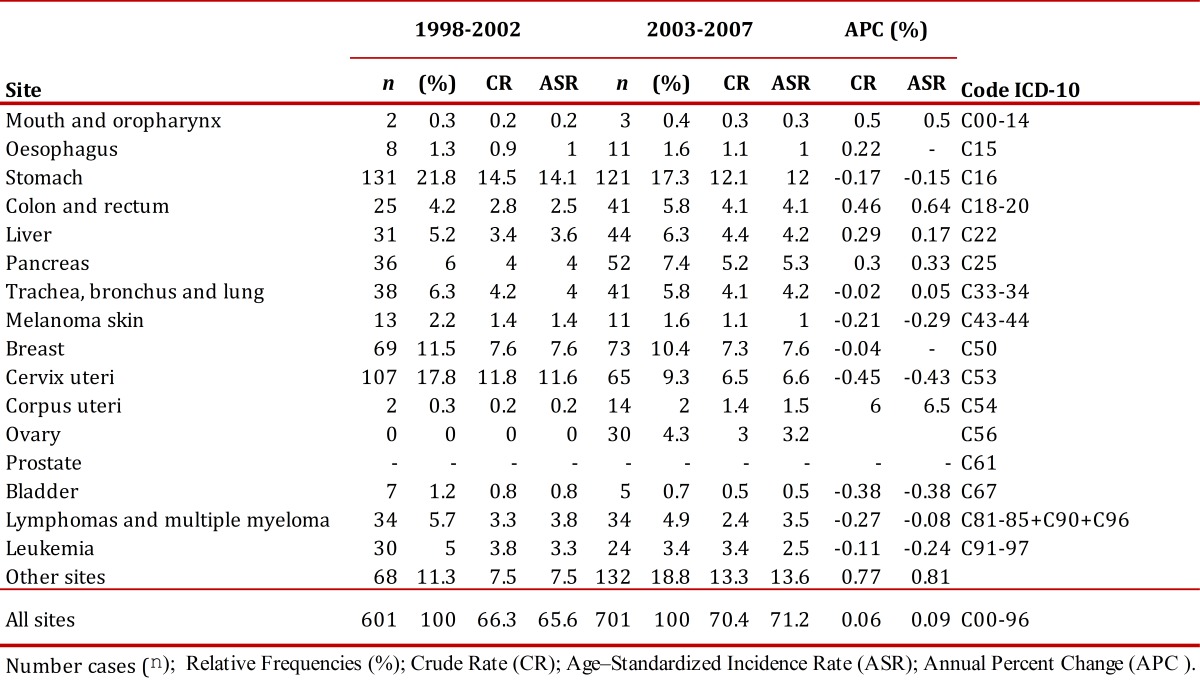
Municipality of Pasto, Colombia. Cancer mortality rates, crude and age-standardized (World standard population) per 100,000 person-years by primary cancer site in females, 1998-2002 and 2003-2007. Number cases (n); Relative Frequencies (%); Rrude Rate (CR); Age - Standardized Incidence Rate (ASR); Annual Percent Change (APC %).

### Cancer incidence and mortality according to sites

**Figure 2:**
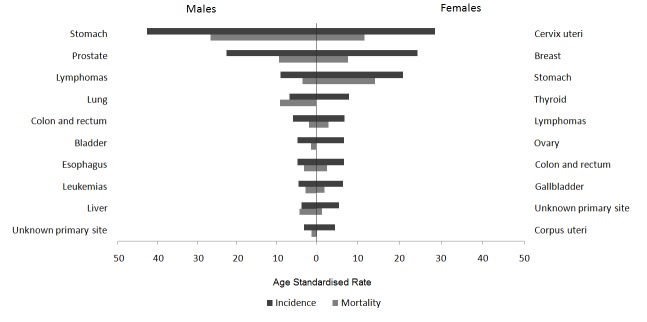
Municipality of Pasto, Colombia. Age- standardized rates per 100,000 person- years of cancer incidence and mortality for the top 10 cancer sites. Males and Females, 1998-2002

**Figure 3.**
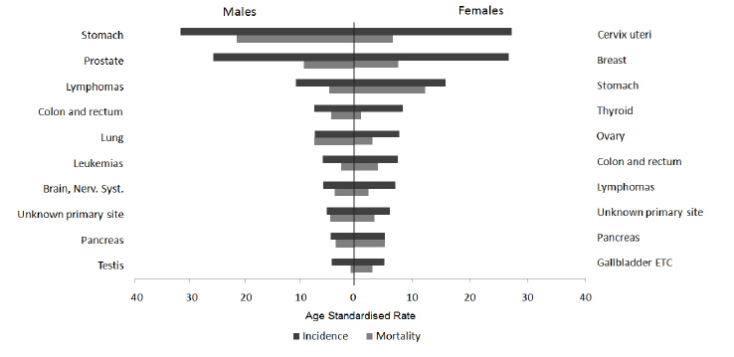
Municipality of Pasto, Colombia. Age- standardized rates per 100,000 person- years of cancer incidence and mortality for the top 10 cancer sites. Males and Females, 2003-2007

For both periods evaluated, Figs. 2 and 3 present the cancer incidence and mortality of the most frequent tumors in the municipality of Pasto according to gender. In males, the most frequent tumors were: stomach (26%), prostate (17%), lymphomas (7%), lung (5%), and colorectal (4.6%). In females, a predominance of cervical (19%), breast (16%), stomach (12%), thyroid gland (6%), and colorectal (5%) tumors is noted. The frequency distribution for the most incidental tumors was kept along the decade evaluated.

In males, stomach tumors represent 32% of deaths due to cancer, followed by lung (11%), prostate (7%), liver (6.7%) tumors and lymphomas (6%). In females, the main causes of death due to cancer were: stomach (19%), cervix (13%), breast (11%), pancreas (7%), and lung (6%). In all, these tumors represent 62% of the total mortality due to cancer in males and 56% in females.

From 2003 to 2007, morbidity and mortality of stomach and cervical cancer diminished, while for the rest of the most frequent tumors in males (prostate, lymphomas, lung, colorectal) and in females (breast, thyroid, colorectal) increased.

During the periods evaluated, the incidence of larynx, esophagus, lung, and bladder tumors was higher in males (GRm/w≈2). Anal, thyroid gland, gall bladder, and breast tumors showed higher incidence rates in females.

### Cancer incidence and mortality by age groups

**Figure 4:**
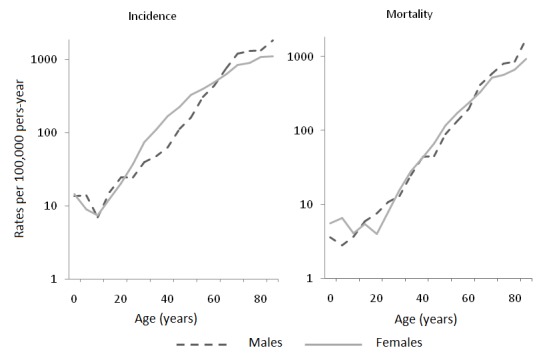
Municipality of Pasto, Colombia. Incidence and mortality rates for all cancers by sex and age, 1998-2007

In general, cancer incidence rates increased with age, mainly as of 30 years of age. Between 25 and 60 years of age, cancer incidence is higher in females than in males. Mortality rates by age groups are similar in males and females (Fig. 4).

The relative frequency of the different types of cancer had variations according to age and gender. The most frequent tumors among individuals younger than 15 years of age were leukemia and lymphomas that represent approximately 62% of the incidence cases and 52% of the deaths in both genders. In the group 15-49 years of age, stomach cancer was in the first place in males (17%), followed by testicular cancer (14%); in females, the highest proportion occurred with cervical cancer (28%) and breast cancer (18%). The main causes of mortality in the same age group were stomach tumors (22%) and lymphomas in males (16%); cervical tumors (24%) and breast tumors (15%) in females.

In the group 50-64 years of age, higher incidence of stomach (30%) and prostate (13%) cancer were reported in males; while breast cancer (23%) and cervical cancer (19%) were highest in females. In this age group, the main causes of mortality are stomach tumors (28%) and lung tumors (12%) in males and stomach tumors (17%) and cervical tumors (17%) in females.

As with the previous group, in the group 65 years and older, stomach cancer (28%) and prostate cancer (25%) in males are the most frequent sites, while in females stomach cancer (19%) appeared as the most incidental, displacing cervical cancer (13%) and breast cancer (11%). Stomach tumors in males (36%) and in females (24%) are the main causes of mortality.

## Discussion

In the municipality of Pasto, when comparing the estimation of population risk, it was found that the annual mean rate of incidence per every 100,000 person-years increased from 133.4 to 138.1 during the periods evaluated. Increased risk of cancer has been documented in different populations around the world and is explained, in part, by population increase and aging, detriment of life habits, broadened coverage of healthcare services, and technological progress as important factors for the detection of new cancer cases^7^.

Aging is a process quite marked in developed nations; it is principally associated to industrialization and economic modernization. Latin American countries are engaged in "full demographic transition", that is, they present a significant fertility decrease and increased life expectancy, favoring the aging of their populations and the increased proportion of elderly adults, who have higher risks of presenting chronic and degenerative diseases like cancer.

The rate between the elderly population and the young population or aging index (AI) for these countries has increased considerably during the last decades, a phenomenon that is also observed in the municipality of Pasto, which for 1985 reported an AI of 9.1 increasing to 23.1 by 2005; a value higher than that reported for Colombia during the same year (16.5)^8^ ^,^ ^9^.

Regarding mortality, in the Municipality deaths due to cancer increased from one period to another, while deaths due to cardiovascular disease - which are the first cause of mortality - diminished. If this tendency were to remain, in the future cancer could represent the first cause of death in the municipality of Pasto.

The 2:1 incidence/mortality proportion, observed in the Municipality, is common in developing countries and differs from that reported for some populations of developed countries, reaching values of 4:1.^10^ These contrasts are mainly explained by the differences in: a) the stage of the disease upon diagnosis; b) access to care and treatment, especially in rural populations; c) offer of healthcare services; d) education, life habits, cultural and psychosocial aspects of the population^11^ ^,^ ^12^.

Regarding the gender ratio, in the municipality of Pasto the risk of developing cancer is 10% higher in females than in males. In industrialized nations, the risk is higher in males and in developing countries it is higher in females. In Quito (Ecuador), it was observed during the last 20 years that the difference between incidence rates per gender is reduced because of an important growth of the rates in males, while in females these have remained stable^13^.

The incidence of tumors associated to cigarette smoking and alcoholism (lung, larynx, esophagus, kidney, and urinary bladder) is higher in males, which suggests higher exposure of the male population from the municipality to these risk factors.^14^ However, the percentage of incidence increase of these tumors during both five-year periods was higher in females. This behavior can be related to changes in the prevalence of cigarette smoking and alcohol consumption in females^15^.

When comparing the incidence rates of the most frequent tumors in the municipality of Pasto during the study periods to those reported for other populations in Latin America, like Cali and Quito, during similar periods, it was found that the population from the municipality is at higher risk of developing stomach cancer in both genders and cervical cancer in females. In breast tumors in females and prostate tumors in males, the risk presented in the municipality is lower to that of these populations^16^.The municipality of Pasto and municipalities from the Andean zone in the Department of Nariño are considered high-risk zones for the development of gastric cancer. Studies conducted in these populations contribute to understanding the process of carcinogenesis, which initiates with a prolonged precancerous stage during childhood as a product of *Helicobacter pylori *infection. The subsequent stages include multifocal gland atrophy, intestinal metaplasia, and dysplasia^17^.

Other studies aimed the knowledge of the dynamics of the *Helicobacter pylori *in children from zones of high and low risk of cancer in the Department of Nariño show that in the municipality of Pasto, considered high risk, the prevalence of the infection in children one year of age was 34.3%, this percentage increased significantly at six years of age to 80%. A similar pattern was observed in Tumaco, with low risk of gastric cancer, where the prevalence was at 32.3% at one year of age and 67.7% at six years of age. The prevalence of *Helicobacter pylori *infection and age at which the bacteria were acquired were similar in both populations, which suggests other determinants of the risk of gastric cancer besides infection, i.e., those associated to the bacteria virulence, external environment, the host and other factors like habits and customs of the individuals^18^.

In populations at high risk of gastric cancer, via effective treatment of* Helicobacter pylori* infection and by complementing the diet with antioxidant micronutrients can interfere in the precancerous process and increase the regression rate of the cancer precursor lesions; this can be an efficient strategy to prevent gastric carcinoma^19^.

In intervened populations in Europe and the United States diminished incidence and mortality of gastric cancer approaches 50% during monitoring periods over 25 years.

Currently, these populations have the lowest rates of gastric cancer incidence and mortality in the world^20^.As with gastric cancer, cervical cancer is considered a disease of multifactor origin and it is associated risk factors: behavioral (number of sex partners, use of oral contraceptives, cigarette smoking) and sexually transmitted infections^21^.

The human papillomavirus (HPV) is considered one of the precursors of the cervical carcinogenesis process; studies show that the HPV is found in approximately 97% of the females with these tumors. The HPV is classified into high- and low-risk strains; the high-risk strains increase by 70% the risk of having cervical cancer^22^.

The WHO and other authors indicate that between 1960 and 2000 cervical cancer prevention programs were successful in populations from developed countries like the USA and Canada, where 60 to 80% reductions were noted in incidence rates and where - currently - the figures remain below 10 annual cases per 100,000 females years^23^.

In Latin America, the Quito Population-based Cancer Registry reported 21% decrease of cervical cancer mortality and incidence during a 10-year evaluation period through the screening program. In Colombia, Cali Cancer Registry identified the cervical cancer epidemic in Cali; this finding served as the basis to establish a vaginal cytology program in the city and documented the drastic decrease of the incidence upon implementing the program^24^.

In the municipality of Pasto, when analyzing the behavior of cervical cancer during both periods a slight decrease of incidence rates was noted compared to the decrease reported in populations with prevention programs.

The rate percentage change of cancer incidence and mortality in the rest of the most frequent tumors in the municipality is coherent with that reported in other populations. In conclusion, cancer in general constitutes an important health problem for the population from the municipality of Pasto.

The global behavior of incidence and increased mortality due to cancer in relationship to other causes of death evidences the need to sponsor and enhance promotion and prevention programs, especially focused on tumors that produce greater morbidity and mortality in the population.
